# Unraveling the induction of phytoene synthase 2 expression by salt stress and abscisic acid in *Daucus carota*

**DOI:** 10.1093/jxb/ery207

**Published:** 2018-05-30

**Authors:** Kevin Simpson, Paulina Fuentes, Luis Felipe Quiroz-Iturra, Carlos Flores-Ortiz, Rodrigo Contreras, Michael Handford, Claudia Stange

**Affiliations:** 1 Laboratorio de Biología Molecular Vegetal, Departamento de Biología, Facultad de Ciencias, Universidad de Chile, Ñuñoa, Santiago, Chile; 2 Laboratorio de Fisiología y Biotecnología Vegetal, Facultad de Química y Biología, Universidad de Santiago de Chile, Santiago, Chile

**Keywords:** Abscisic acid, carrot, DcAREB transcription factors, DcPSY2, salt stress

## Abstract

Phytoene synthase (PSY) is the first committed enzyme of the carotenoid biosynthesis pathway and the most important point of regulation. Carotenoids are precursors of abscisic acid (ABA), which mediates abiotic stress tolerance responses in plants. ABA activates the synthesis of its own precursors through induction of *PSY* expression. Carrot, a species that accumulates very high amounts of carotenoids in its reserve root, has two *PSY* paralog genes that are expressed differentially in the root. Here, we determined that *DcPSY2* expression is induced by salt stress and ABA. A *DcPSY2* promoter fragment was obtained and characterized. Bioinformatic analysis showed the presence of three ABA responsive elements (ABREs). Through overexpressing pPSY2:GFP in *Nicotiana tabacum* we determined that all three ABREs are necessary for the ABA response. In the carrot transcriptome, we identified three ABRE binding protein (DcAREB) transcription factor candidates that localized in the nucleus, but only one, DcAREB3, was induced under ABA treatment in carrot roots. We found that AREB transcription factors bind to the carrot *DcPSY2* promoter and transactivate the expression of reporter genes. We conclude that *DcPSY2* is involved in ABA-mediated salt stress tolerance in carrot through the binding of AREB transcription factors to its promoter.

## Teaser

ABA increased during salt stress triggers binding of AREB/ABFs to *DcPSY2* promoter, boosting carotenoid and ABA levels as a positive feedback mechanism

## Introduction

Carotenoids are colored lipid-soluble molecules that act as accessory pigments and have photoprotective functions during photosynthesis (Telfer, 2005; Stange and Flores, 2012). They also protect cells from excessive light energy through thermal dissipation and supply substrates for the biosynthesis of the plant growth regulator abscisic acid (ABA) (Crozier *et al.*, 2000) and of strigolactones (Moreno Beltran and Stange, 2016). Carotenoids also play an important role in human nutrition and health, providing provitamin A, having anti-aging and anti-cancer activities, and preventing age-related macular degeneration (Mayne, 1996; Krinsky and Johnson, 2005; Rao and Rao, 2007), which has resulted in the development of various nutraceutical products containing carotenoids.

**Fig. 1.**
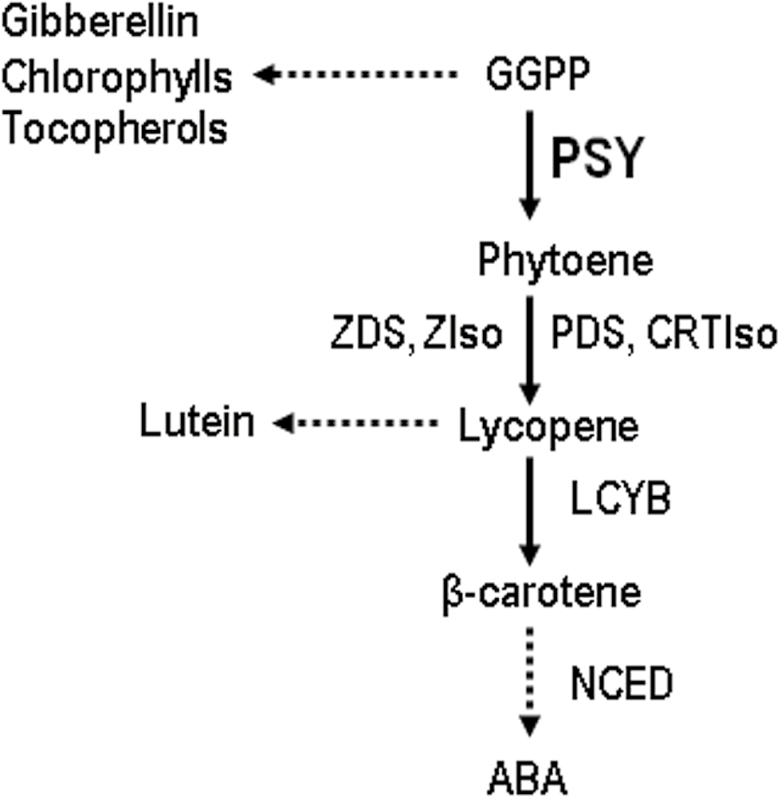
Summary of the main steps in carotenoid and ABA synthesis. Dashed arrows represent several enzymatic steps. ABA, abscisic acid; CRTIso, carotene *cis–trans* isomerase; GGPP, geranylgeranyl pyrophosphate; LCYB, lycopene β-cyclase; NCED, 9-*cis*-epoxycarotenoid dioxygenase; PDS, phytoene desaturase; PSY, phytoene synthase; ZDS, ζ-carotene desaturase; ZIso: ζ-carotene isomerase.

Carotenoid synthesis takes place in plant plastids and is achieved by several enzymes in a highly coordinated mechanism. Among these, phytoene synthase (PSY) is the first enzyme of the pathway (Fig. 1) and the most important point of regulation (von Lintig *et al.*, 1997; Lois *et al.*, 2000; Li *et al.*, 2008; Rodríguez-Villalón *et al.*, 2009*a*; Rosas-Saavedra and Stange, 2016). In Arabidopsis, *PSY* is present in a single copy, but most economically important crops, such as *Solanum lycopersicum* (tomato), *Zea mays* (maize), *Nicotiana tabacum* (tobacco), *Oryza sativa* (rice), *Daucus carota* (carrot) and *Manihot esculenta* (cassava), have several paralogous *PSY* genes (Bartley and Scolnik, 1993; Busch *et al.*, 2002; Just *et al.*, 2007; Li *et al.*, 2008; Welsch *et al.*, 2008; Arango *et al.*, 2010).

Plants have developed diverse mechanisms for regulating carotenoid synthesis and accumulation (Lu and Li, 2008). The synthesis of carotenoids can be regulated by sequestration and accumulation in different types of plastids (Deruère *et al.*, 1994; Vishnevetsky *et al.*, 1999), at the post-translational level and even at the epigenetic level (Cazzonelli *et al.*, 2009). Nevertheless, one of the most prevalent and important mechanisms is the regulation of the expression of genes involved directly in the synthesis of carotenoids (Cazzonelli and Pogson, 2010), which is influenced by external and developmental factors such as light, fruit ripening, biotic and abiotic stress, negative–positive feedback mechanisms, and in response to hormones (Lu and Li, 2008).

ABA is synthesized in the cytoplasm through the carotenoid pathway (Fig. 1), and is a hormone that participates in many physiological processes (Zeevaart and Creelman, 1988) such as seed dormancy, regulation of plant growth, senescence, control of stomatal aperture, and tolerance to abiotic stresses such as cold, drought, and salinity. (Leung and Giraudat, 1998; Finkelstein *et al.*, 2002; Himmelbach *et al.*, 2003; Yamaguchi-Shinozaki and Shinozaki, 2005; Daszkowska-Golec and Szarejko, 2013). Although it has been observed in citrus that the accumulation of ABA in dehydrated roots depends on transport from aerial organs (Manzi *et al.*, 2015, 2016), *de novo* synthesis of ABA has been reported during osmotic stress in many plant models, with 9-*cis*-epoxycarotenoid dioxygenase (NCED) constituting a limiting step in stress-induced biosynthesis (Schwartz *et al.*, 1997; Thompson *et al.*, 2000). This suggests that an increase in carotenoid synthesis is necessary for elevating the levels of ABA to respond to osmotic stress in plant roots (Audran *et al.*, 1998; Thompson *et al.*, 2000, 2007; Arango *et al.*, 2010; Ruiz-Sola and Rodríguez-Concepción, 2012). In this sense, ABA regulates the synthesis of its own metabolic precursors by inducing the expression of carotenogenic genes. In Arabidopsis, osmotic and salt stresses, as well as the exogenous application of ABA, induce the expression of *PSY* in the root, leading to an increase in carotenoid accumulation (Meier *et al.*, 2011; Ruiz-Sola *et al.*, 2014). In maize and rice, only the *PSY3* paralog is induced by salt and ABA in the root, while *PSY1* and *PSY2* are induced by light in leaves (Li *et al.*, 2008, 2009; Welsch *et al.*, 2008). This indicates that *PSY* paralogs can exhibit functional specificity and diversity in plants.

One of the most important physiological responses mediated by ABA is achieved through the regulation of gene expression (Busk and Pagès, 1998). Promoter analysis of genes regulated by ABA reveal that the conserved sequence ACGTGG/TC is a determining *cis*-element for ABA response, and was denominated the ABA responsive element (ABRE; Busk and Pagès, 1998; Hattori *et al.*, 2002; Yoshida *et al.*, 2010). This element belongs to the G-box family, which has been implicated in a broad range of processes in plants (Menkens *et al.*, 1995), and was first identified in the promoters of the *Em* and *rab-16A* genes (Marcotte *et al.*, 1989; Mundy *et al.*, 1990). The ABRE contains the central sequence ACGT, which is recognized by several bZIP transcription factors (Guiltinan *et al.*, 1990; Hobo *et al.*, 1999; Choi *et al.*, 2000; Uno *et al.*, 2000). One copy of the ABRE is not enough to activate the transcription mediated by ABA; a second ABRE close by (Guiltinan *et al.*, 1990; Hobo *et al.*, 1999; Choi *et al.*, 2000; Uno *et al.*, 2000) or a coupling element is required to establish the ABA response complex (ABRC; Shen *et al.*, 1996). Most coupling elements are similar to the ABRE and contain the A/GCGT motif.

The bZIP transcription factors that recognize the ABRE are called ABA-responsive element binding proteins (AREBs; Uno *et al.*, 2000) or ABRE-binding factors (ABFs; Choi *et al.*, 2000). Among the nine AREB/ABFs described to date in Arabidopsis, AREB1/ABF2, AREB2/ABF4, ABF1, and ABF3 are induced by ABA and abiotic stress in vegetative tissues (Choi *et al.*, 2000; Uno *et al.*, 2000; Kang *et al.*, 2002; Kim *et al.*, 2002; Fujita *et al.*, 2005; Furihata *et al.*, 2006; Kim, 2006). The *areb1*/*areb2*/*abf3* triple knockout mutant has impaired expression of ABA- and osmotic stress-responsive genes, together with an increase in sensitivity to drought and a decrease in ABA sensitivity in primary root growth. Therefore, AREB1, AREB2, and ABF3 have been considered as master transcription factors in ABA signaling involved in drought stress tolerance.

*Daucus carota* L. var. *sativus* (2*n*=18) is a biennial plant that belongs to the botanical group Apiaceae (or Umbelliferae). Carrot is one of the 10 most important vegetables cultivated worldwide and one of the few plants that synthesize and accumulate massive amounts of carotenoids in the storage root (Just et al., 2007, 2009). In carrot, two *PSY* paralog genes have been identified (Just *et al.*, 2007, 2009). Both genes are expressed differentially during carrot development. *DcPSY1* is expressed preferably in leaves while *DcPSY2* expression is higher during carrot storage root development (Fuentes *et al.*, 2012). Moreover, when comparing orange and wild white (Ws) carrot inbred lines, the expression of *DcPSY2* correlates more strongly than that of *DcPSY1* with the accumulation of carotenoids in roots of the orange cultivar (Wang *et al.*, 2014). Overexpression analyses showed that both *DcPSY1* and *DcPSY2* are functional, resulting in increased carotenoid accumulation in transgenic carrots (Flores personal communication). In addition, PSY activity has been proven to be a limiting step in carotenoid synthesis in carrot roots, as the overexpression of *PSY* is sufficient to produce carotenoids in a white variety (Maass *et al.*, 2009). However, at present no information on the regulation of *PSY* genes by ABA or abiotic stress has been reported in carrot. In this study, we determined that *DcPSY2* and *DcPSY1* are strongly up-regulated in response to salt in leaves and roots of carrot seedlings in correlation with an increase in ABA. However, only *DcPSY2* expression is induced by ABA in carrot roots, which is associated with the presence of ABREs in its promoter. Moreover, we analysed the binding and ABA response of three carrot ABRE-binding factors (AREB/ABFs) and determined that DcAREB3 is most likely the mediator of *DcPSY2* induction under salt stress via ABA.

## Materials and methods

### Plant material

Seeds of commercially acquired carrot (*Daucus carota* L.) cultivar Nantaise and seeds of tobacco (*Nicotiana tabacum* var Xanthi NN) were surface sterilized in a solution of 95% ethanol for 1 min and washed once with sterile water for 3 min. Then, carrot seeds were incubated under agitation in a solution of sodium hypochlorite (2.62% v/v) for 45 min, washed three times with sterile water and finally dried on sterile absorbent paper. Tobacco seeds were incubated for 15 min in sodium hypochlorite (2.62% v/v). All seeds were deposited in sterile flasks with solid MS medium (Murashige and Skoog, 1962) supplemented with 4.4 g l^−1^ MS salts, 0.44% vitamins, 2% sucrose, 0.01% *myo*-inositol, and 0.7% agar (pH 5.8), and kept in a growth chamber with a 16 h day photoperiod illuminated with cool-white fluorescent light (115 μmol m^−2^ s^−1^) at 22 °C for 4–6 weeks.

### Genome walking

The GenomeWalker (Clontech) methodology was used to obtain the *DcPSY2* promoter of *D. carota* (Siebert *et al.*, 1995). Carrot genomic DNA of high purity, obtained by the CTAB method, was digested with *Dra*I, *Eco*RV, *Pvu*II, and *Stu*I blunt-ended endonucleases and ligated to an adapter sequence (AP) following the manufacurer’s instructions. Four digestion libraries (BD1, BD2, BD3, and BD4) were produced with the enzymes *Dra*I, *Eco*RV, *Pvu*II, and *Stu*I, respectively. The first PCR amplification (primary PCR) was carried out in a volume of 25 µl containing nuclease-free water, 1× Taq DNA polymerase buffer (Bioline), 2 U Taq DNA polymerase (Bioline), 3 mM MgCl_2_, 200 µM dNTPs, 200 nM AP-specific binding linker (AP1), and 200 nM of an antisense primer specific for the *DcPSY2* gene, GW1PSY2 (see Supplementary Table S1 at *JXB* online). One microliter of each digestion library was used as sample in each independent reaction (GW1 PCR). For the nested PCR (secondary PCR), 1 μl of a 1/50 dilution of the primary PCR (GW1 PCR) was used. In this case, the AP2 (Supplementary Table S1) sense primer, an AP-specific junction primer that is located downstream of the AP1 primer, and the GW2*PSY2* gene-specific antisense primer located upstream of the *DcPSY2* primary primer were used (GW2 PCR). The GW2 PCR products were visualized by electrophoresis in agarose gels and the bands of interest (greater than 500 bp) were purified from the gel for subsequent cloning and sequencing. We obtained a promoter fragment of 769 bp that was cloned and sequenced (Supplementary Fig. S1) for further characterization.

### Vector construction

For *DcPSY2* promoter characterization, P1 (421 bp) and P2 (769 bp) fragments of the *DcPSY2* promoter were amplified using primers P1-PSY2R and P2-PSY2R, respectively (see Supplementary Table S1). P1 and P2 sequences were cloned into pCR8/GW/TOPO (Invitrogen) following the manufacturer’s instructions. Positive clones obtained by enzymatic digestion were sequenced by Macrogen Corp. (USA). Subsequently, pCR8/P1 and pCR8/P2 were recombined into pMDC111 to produce the pMDC111/P1::GFP and pMDC11/P2::GFP expression vectors. Positive clones were analysed through PCR and enzymatic digestion with *Eco*RI and *Hin*dII for P1 and *Bst*EII and *Ssp*I for P2, and transformed into *Agrobacterium tumefaciens* (GV3101 strain). These constructions were used for stable tobacco transformation.

For AREB1, AREB3, and AREB4 subcellular localization, the complete coding sequences without the stop codon were amplified using primers listed in Supplementary Table S1 and cloned into pCR8/GW/TOPO (Invitrogen) following the manufacturer’s instructions. Positive clones obtained by enzymatic digestion were sequenced by Macrogen Corp. (USA). Subsequently, pCR8/AREBs were recombined into pk7RWG2 to produce the pk7RWG2/AREBs expression vectors in which each gene is fused at the 3′ end with red fluorescent protein (RFP) to produce the AREB:RFP fusion proteins. Positive clones were analysed through PCR and enzymatic digestion, and transformed into *Agrobacterium tumefaciens* (GV3101 strain).

For monohybrid and transactivation assays, the P2 promoter was cloned into pAbAi in which the promoter directs the expression of the antibiotic resistance gene *AurobasidinA*. Primers with *Sac*I and *Sal*I restriction sites were synthesized to amplify the P2 promoter from the pCR8/P2 vector. The amplified P2 fragment was digested with *Sac*I and *Sal*I and cloned into pAbAi, to produce the pP2-pAbAi vector. Positive clones were analysed through PCR and enzymatic digestion. Subsequently, the pCR8/AREB vectors were recombined into the pDEST22 and pDEST32 vectors (Invitrogen) to produce pDEST22/AREBs (AREB transcription factors fused to the GAL4 activation domain) and pDEST32/AREBs (AREB transcription factors fused to the DNA binding domain) vectors.

### Tobacco transformation

Tobacco seedlings grown for 6 weeks in solidified MS medium were transformed according to a previous report (Horsch *et al.*, 1985). Briefly, leaf explants were co-cultivated with *Agrobacterium* carrying pMDC111/P1::GFP or pMDC111/P2::GFP, and placed on solidified MS medium containing 1 mg l^−1^ 6-benzylaminopurine, 0.5 mg l^−1^ indole-3-butyric acid, 25 mg l^−1^ kanamycin and 300 mg l^−1^ cefotaxime. After 4 weeks, the explants were placed on solidified MS medium supplemented with 50 mg l^−1^ kanamycin and 100 mg l^−1^ cefotaxime in the absence of hormones to induce the development of transgenic plantlets. When plantlets with proper root development reached 7 cm in height, they were transferred to plastic pots (20 × 10 cm) containing a mix of soil and vermiculite (2:1). Transgenic T0 plants were analysed by PCR and RT-PCR and five to seven lines were selected for further characterization of the P1 and P2 promoters.

### Salt and ABA treatments

For ABA treatment, leaf samples of transgenic tobacco bearing the constructs pMDC111/P1::GFP (five T0 lines) or pMDC111/P2::GFP (seven T0 lines) were collected and divided into four equal parts. Each part was incubated in sterile liquid MS medium supplemented with 3% sucrose and ABA at concentrations of 0, 50, 100, and 200 µM (Kiyosue *et al.*, 1992;Chung *et al.*, 2005) for 24 h in dark at 22–25 °C with gentle shaking. The experiment was performed in duplicate for each transgenic line, and after 24 h, *GFP* expression was analysed by qRT-PCR. The results were expressed taking each transgenic line as a biological replicate.

For NaCl treatment of *D. carota*, groups of fifteen 4-week-old plants were used. These were incubated in 250 mM NaCl solution for 2 h. At the end of this period, leaf and root tissues were collected for *DcPSY1* and *DcPSY2* qRT-PCR analysis. As control, distilled water was used. The assay was repeated twice. For ABA treatment of *D. carota*, pools of fifteen 4-week-old plants were used. The plants were incubated in 100 μM ABA for 2, 4, and 6 h. At the end of these periods, leaf and root tissues were collected for *DcPSY1*, *DcPSY2*, *AREB1*, *AREB3*, and *AREB4* qRT-PCR analysis. As control of the treatments, distilled water was used. The assay was repeated twice with three technical replicates each.

### ABA quantification

A pool of 15 carrot seedlings cultivated for 4 weeks in full-strength MS medium supplemented with 1% sucrose were taken for acute salt treatment for 2, 4, and 6 h. Two hundred milligrams of leaves and root tissue obtained were flash frozen and ground with a mortar and pestle in the presence of liquid N_2_ and 4 ml of H_2_O milli-Q. The mixture was shaken at 4 °C for 20 min and incubated over night at 4 °C. Afterwards, the samples were centrifuged at 18 000 *g* for 15 min, and the aqueous phase containing ABA was recovered. ABA determination was performed using an HPLC–electrospray ionization tandem mass spectrometry system (Agilent 1200 series, MS/MS5420, Agilent Technologies, Santa Clara, CA, USA) following the instructions described (González *et al.*, 2014). The mobile phase was composed of 0.1% formic acid. A sample of 20 µl was separated using a C18 reverse phase column with a flow rate of 0.3 ml min^−1^ at room temperature. The experiment was carried out in duplicate.

### RNA extraction and quantitative RT-PCR

Total RNA was extracted from frozen powder of transgenic tobacco leaves and carrot leaves and roots using RNAsolv (Omega Biotec, USA). Genomic DNA traces were eliminated by a 20 min DNaseI treatment. For cDNA synthesis, 2 μg total DNA-free RNA was mixed with 1 mM oligo AP (5′-CGCCACGCGTCGACT AGTACTTTTTTTTTTTTTTTTT-3′) and Impron II reverse transcriptase (Promega). qRT-PCR experiments were performed in a LightCycler system (Stratagene), using SYBR Green double strand DNA binding dye, as described (Fuentes *et al.*, 2012). Specific primers targeting the coding region of *DcPSY1*, *DcPSY2*, *DcAREB1*, *DcAREB3*, *DcAREB4*, and *DcOsmotin* were used (see Supplementary Table S1). *DcUbiquitin* and *Nt18S* were used as housekeeping genes for *D. carota* and *N. tabacum*, respectively. The relative expression levels of each gene in the different conditions were calculated using the crossing point values and the equation described in Pfaffl (2001). Each qRT-PCR reaction was performed with three biological and two technical replicates. In all cases, the reaction specificities were tested with melting gradient dissociation curves and electrophoresis gels.

### Carotenoid quantification

Carotenoids from a pool of fifteen 4-month-old wild type carrot seedlings were extracted from 100 mg of leaves and roots with 1 ml hexane/acetone/ethanol (2:1:1 v/v) as described (Fuentes *et al.*, 2012). Two successive extractions were performed to remove carotenoids until the tissue was blanched. The extract was dried with gaseous nitrogen and resuspended in 1 ml acetone. Total carotenoids were measured by spectrophotometry at 474 nm. The samples were maintained on ice and in the dark to avoid photo-degradation, isomerization and/or structural changes of pigments.

### Subcellular localization

The pK7RWG2/AREB vectors (which encode the chimeric AREB:RFP proteins) and pCAMBIA1302 construct (CaMV35S::GFP) were transiently expressed in leaves of 2-month-old *Nicotiana tabacum* plants by agroinfiltration (Utz and Handford, 2015). The samples were visualized in an inverted epifluorescence microscope (IX-70, Olympus America Inc., Melville, NY, USA). The images were taken for each sample with a ×40 magnification. GFP and RFP fluorescence images were taken using an excitation light of 450–490 nm (blue light) and 530–560 nm (green light), respectively. Images were processed with LSM5 Image Browser and Adobe Photoshop software.

### Protoplast isolation and transfection

*Daucus carota* protoplast isolation was carried out adapting the protocol of (Moscatiello *et al.*, 2013). Briefly, 50 ml of 4- to 5-day-old *D. carota* cell suspensions were centrifuged at 200 *g* for 2 min. The supernatant was removed and 10 ml of enzyme solution (Cellulase Onozuka R-10 0.75% w/v, 0.25% macerozyme R-10, 400 mM mannitol, 10 mM CaCl_2_, 2.5 mM MES, pH 5.7) was added. The cells were transferred to Petri dishes and incubated in the dark on an orbital shaker (40–50 rpm) for 4–6 h. The cell suspension was filtered through a 40 μm nylon membrane, and the protoplasts were centrifuged at 60 *g* for 5 min. The supernatant was removed by aspiration and the protoplasts were washed twice in W5 solution (154 mM NaCl, 5 mM KCl, 125 mM CaCl_2_, 5 mM glucose, pH 5.7) and incubated in W5 solution on ice for 30 min. Then, the protoplasts were centrifuged at 60 *g* for 5 min and resuspended in MC solution (5 mM MES, 20 mM CaCl_2_, 0.5 mM mannitol, pH 5.7). Finally, protoplasts were counted under an optical microscope using a Neubauer chamber and resuspended to a concentration of 2 × 10^6^ protoplasts ml^−1^. The transfection of *D. carota* protoplasts was performed according to the protocol of Liu *et al.* (1994); 300 μl of protoplasts in MC solution (2 × 10^6^ protoplasts ml^−1^) were mixed with 10–20 μg of plasmid DNA (each pK7RWG2/AREB vector) and 300 μl of 40% PEG solution (40% w/v polyethylene glycol, 100 mM Ca(NO_3_)_2_, 400 mM mannitol, pH 10). Samples were incubated for 5 min and then 4 ml of PSM solution (MS medium with vitamins 0.44% w/v, sucrose 2% w/v, pH 5.7) was added. Finally, protoplasts were incubated for 18–24 h in darkness at room temperature before being visualized under an inverted epifluorescence microscope (Olympus IX70 using the filter fluorescein isothiocyanate for GFP (450–490 nm) and Cy3 for RFP (530–560 nm)).

### Bioinformatic analysis of the *DcPSY2* promoter

Bioinformatic analysis of the *DcPSY2* promoter region was performed in the TOUCAN2 program (http://homes.esat.kuleuven.be/~saerts/software/toucan.php). To identify regulatory motifs, the MotifScanner bioinformatics tool (http://homes.esat.kuleuven.be/~sistawww/bioi/thijs/download.html) was used. As a position weight matrices (PWM) database, we used the PlantCARE Database (http://bioinformatics.psb.Ugent.be/webtools/plantcare/html/), which contains 435 regulatory motifs for mono- and dicotyledonous plants. Because the genome of *D. carota* was not sequenced at the time of performing this anaylsis, we used the Arabidopsis genome as the background model, the only third-order plant organism within the candidate list, as recommended (http://toucan.aertslab.org/software/toucan.php#man). A ‘prior’ value of 0.7 was established (*a priori* probability of finding a motif) and only the positive strand of DNA was analysed. The transcription initiation site was defined using the BDGP bioinformatic program (http://www.fruitfly.org/seq_tools/other.html). According to the distribution of the ABRE motifs found and considering the transcription initiation site, P2 and P1 promoter fragments of *DcPSY2* were defined. The bioinformatic tool Standalone BLAST, the database of plant transcription factors PlantTFDB (http://planttfdb.cbi.pku.Edu.cn/index.php;Jin *et al.*, 2014), and the sequences present in the transcriptome of *D. carota* (Iorizzo *et al.*, 2011) were used to find AREB/ABF transcription factors in *D. carota*. To analyse conserved domains in the AREB/ABF transcription factors, the NCBI CD-Search tool (http://www.ncbi.nlm.nih.gov/Structure/cdd/wrpsb.cgi) was used (Marchler-Bauer *et al.*, 2015).

### Monohybrid and transactivation assay

The monohybrid assay was performed through the Matchmaker® Gold Yeast One-Hybrid Library Screening System (Clontech, kindly provided by Dr Raúl Herrera, University of Talca). Yeast Y1HGold strains containing the pP2-AbAi vector (Y1HGold-pP2-AbAi strain), in which the aurobasidin A (AbAi) resistance gene is directed by the *DcPSY2* promoter, were generated. The determination of the minimum inhibitory concentration of AbAi was performed according to the manufacturer’s instructions (Clontech/Takara Bio USA, Inc.). For binding assays of AREB transcription factors to the P2 promoter of *DcPSY2*, the Y1HGold-pP2-AbAi strain was transformed with the pDEST22/AREB vectors. Subsequently, the yeasts were grown in SD/-Trp-Ura medium. The binding was measured as the capability of the yeast strains transformed with *DcAREB1*, *DcAREB3*, and *DcAREB4* to grow in the AbAi medium. For transactivation assays, the pDEST32/AREB (ABRE2, ABRE3, and ABRE4 fused to the GAL4 DNA binding domain) vectors were transformed into the yeast strain MaV203 (Vidal *et al.*, 1996), and grown in SD/−Leu medium. The MaV203 strain possesses three stably integrated GAL4 inducible reporter genes: *HIS3*, *URA3*, and *lacZ*. Thus, the transactivation of the reporter genes was measured as the capability of the yeast strains to grow in the absence of histidine and uracil. In addition, induction of the *lacZ* gene resulted in blue colored colonies when tested with 5-bromo-4-chloro-3-indolyl-β-D-galactopyranoside (X-gal).

## Results

### Salt stress induces the expression of *DcPSY1* and *DcPSY2* and the synthesis of ABA in carrot leaves and roots but only *DcPSY2* expression is induced by ABA

**Fig. 2.**
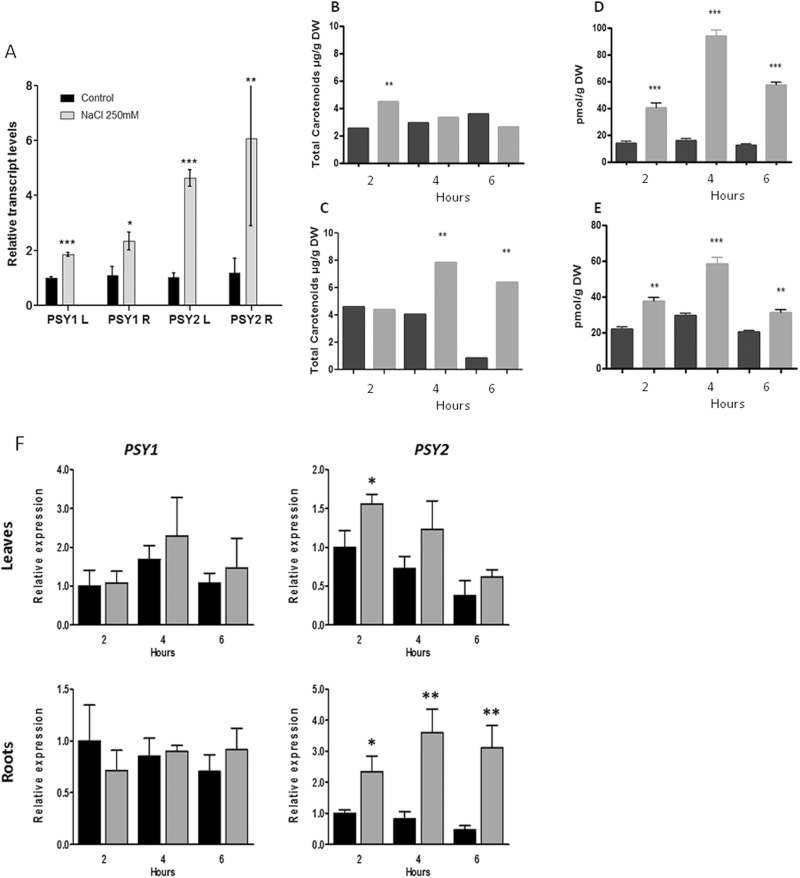
Relative expression of *DcPSY1* and *DcPSY2* under salt and ABA treatment. (A) Normalized transcript levels of *DcPSY1* and *DcPSY2* in leaves (L) or roots (R) of 4-week-old carrot plants after 2 h of water (black bars) or 250 mM NaCl treatment (gray bars). (B, C) Carotenoid quantification in leaves (B) and roots (C) in 4-week-old carrot plants after 2, 4, and 6 h of water (black bar) or 250 mM NaCl treatment (gray bar). (D, E) ABA quantification in leaves (D) and roots (E) in 4-week-old carrot plants after 2, 4, and 6 h of water (black bars) or 250 mM NaCl treatment (gray bars). (F) Normalized transcript levels of *DcPSY1* and *DcPSY2* in leaves or roots of 4-week-old carrot plants after 2, 4, and 6 h of water (black bars) or 100 µM ABA treatment (gray bars). Transcript abundance was normalized to *ubiquitin* expression level and the control condition was taken as calibrator. All values represent the means of three independent values (+SD). Asterisks indicate statistically significant differences: *P < 0.05, **P < 0.001.

In order to determine if the *PSY* paralog genes in carrot respond differentially to abiotic stress, as was reported previously in other species, 4-week-old carrot seedlings were treated with 250 mM NaCl and the expression levels of *DcPSY1* and *DcPSY2* were measured in leaves and roots. Figure 2A shows that the expression levels of *DcPSY1* and *DcPSY2* increased significantly after NaCl treatment in both organs. Although salt stress did not produce changes in carotenoids in leaves of treated seedlings, it resulted in an increase of total carotenoids in roots at 4 and 6 h (Fig. 2B, C) and a significant induction of ABA levels in both, leaves and roots (Fig. 2D, E). These results suggest that salt stress induces an increase of ABA in leaves and roots of carrot through the expression of both *PSY* paralogs of *D. carota*. Interestingly, the increase in the expression levels of *DcPSY2* was greater in both organs (6-fold in the root and 6-fold in the leaves) than the increase of *DcPSY1* (2.2-fold in the root and 2-fold in the leaves), suggesting that *DcPSY2* plays a more prominent role in this abiotic stress response, indicating a differential role for both *DcPSY* paralogs, as has been observed in maize and rice (Li *et al.*, 2008; Welsch *et al.*, 2008).

The Arabidopsis *PSY* gene that is induced by salt stress is also directly induced by ABA (Ruiz-Sola *et al.*, 2014). To determine if the increase in the expression levels of *DcPSY1* and/or *DcPSY2* could be directly mediated by ABA, 4-week-old carrot seedlings were treated with 100 μM ABA for 2, 4, and 6 h. The exogenous application of ABA significantly increased transcript levels of *DcPSY2*, but not *DcPSY1* (Fig. 2F), mainly in carrot roots at all periods of treatment. This result suggests that ABA regulates the synthesis of its own metabolic precursors through the induction of the expression of *DcPSY2* in *D. carota* and is consistent with findings reported in other plant models where different *PSY* paralogs have functional specificity (Li *et al.*, 2008, 2009; Welsch *et al.*, 2008).

### Identification and characterization of the *DcPSY2* promoter of *Daucus carota*

**Fig. 3.**
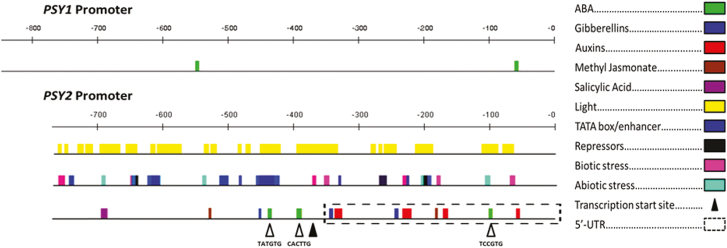
Regulatory elements in *DcPSY2* promoter by *in silico* analysis. The 769 bp promoter *DcPSY2* fragment obtained in this work presents different regulatory elements. The predicted ABREs are represented in the *DcPSY1* and *DcPSY2* promoter taken from our sequence analysis and from the carrot genome (Iorizzo *et al.*, 2016).

Taking into account that the regulation of the carotenoid biosynthetic pathway occurs mainly at the transcriptional level, in particular the regulation of *PSY* expression (Li *et al.*, 2008; Rodríguez-Villalón *et al.*, 2009*b*; Toledo-Ortiz *et al.*, 2010; Welsch *et al.*, 2008), and considering that ABA activates the expression of *DcPSY2* in carrot, we isolated the promoter of *DcPSY2* in order to identify possible ABREs. By means of genome walking we obtained a 769 bp region of the *DcPSY2* promoter that was cloned and sequenced (see Supplementary Fig. S1). The search for predicted regulatory motifs in the *DcPSY2* promoter was performed using the bioinformatics tool TOUCAN 3.1.1 and the results are shown in Fig. 3. Several of the identified regulatory motifs correspond to light responsive elements, as well as to phytohormones (auxins, gibberellins, among others) and to certain types of abiotic (drought, low temperature, anaerobiosis) and biotic (pathogens, elicitors) stresses (Fig. 3; Supplementary Table S2). Interestingly, within the *DcPSY2* promoter region, three ABREs were identified, two of which are in close proximity to each other (27 bp) (Fig. 3). The sequence of the promoter region obtained, as well as the presence of the ABRE motifs, is consistent with those found in the recently sequenced carrot genome (Iorizzo *et al.*, 2016), supporting our findings and the further study of this promoter fragment. Considering that an ABRE must be associated to another ABRE or to a coupling element (Shen *et al.*, 1996; Hobo *et al.*, 1999), the presence of two ABREs close to each other suggests that they could constitute an ABRC (Shen *et al.*, 1996; Gómez-Porras *et al.*, 2007). The same bioinformatics analysis performed on the *DcPSY1* promoter taken from the genomic and transcriptomic database for carrot (CarrotDB; Xu *et al.*, 2014) revealed that, although this promoter possesses two ABREs (Fig. 3), they are distant from each other, which could explain the lack of transcriptional activation in response to ABA treatments (Fig. 2F).

**Fig. 4.**
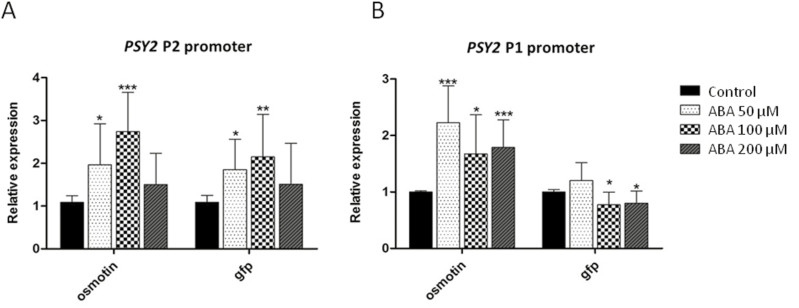
Characterization of *DcPSY2* promoter through stable expression in *N. tabacum* plants. Relative expression of *GFP* in DcPSY2P2:GFP and DcPSY2P1:GFP transgenic lines under ABA and water (control) treatments. Measurements correspond to the mean of five to seven independent T0 transgenic plants. Each plant was analysed separately with three biological replicas and two technical replicas. Data were normalized to *NtRNA18S* and the control condition was used as calibrator. Asterisks denote significant differences in gene expression between a determined ABA concentration and the respective control, using an unpaired two-tailed *t*-test. *P < 0.05, **P < 0.01, ***P < 0.001.

To determine whether the ABREs predicted in the *DcPSY2* promoter are involved in the ABA response, we generated transgenic *N. tabacum* plants carrying two different versions of the *DcPSY2* promoter directing the expression of *GFP*. In tobacco transgenic lines carrying the longest promoter version (P2 promoter), which contains all three ABREs (Fig. 3), an increase in the expression levels of *GFP* was observed after the exogenous application of different concentrations of ABA (Fig. 4A), whereas no increase in *GFP* expression levels was observed in tobacco plants carrying the P1 promoter, which contains only the two distant ABREs, after the application of ABA (Fig. 4B). The expression levels of *osmotin*, a well described ABA target gene (Fujita *et al.*, 2005), were monitored as a control for the ABA treatment. These results show that the *DcPSY2* promoter of *D. carota* is able to respond to ABA, and suggest that this response depends on the presence of at least two ABREs in close proximity.

### Identification and functional characterization of AREB/ABF transcription factors in *D. carota*

**Fig. 5.**
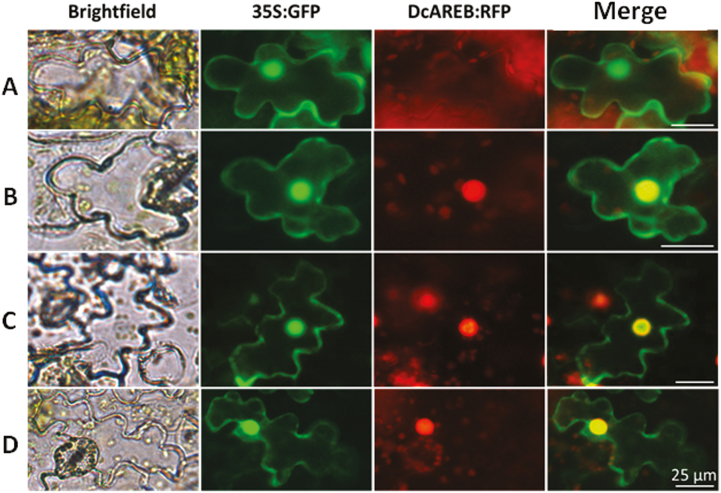
Subcellular localization of DcAREB transcription factors. Co-transformation of tobacco leaves with *Agrobacterium* carrying the DcAREB:RFP vectors and the 35S:GFP vector as a known nuclear localization marker. Images were taken 4 days after tobacco leaf infiltration. (A) pK7RWG2 empty vector carrying the 35S:RFP construct; (B) DcABRE1:RFP; (C) DcABRE3:RFP; (D) DcABRE4:RFP. Red channel: images taken under the Cy3 filter. Green channel: images taken under the fluorescein isothiocyanate filter. Scale bar: 25 μm.

To identify possible transcription factors that bind and transactivate the *D. carota DcPSY2* promoter in response to ABA, we performed an alignment between the transcriptome of *D. carota* (Iorizzo *et al.*, 2011) and the database of plant transcription factors PlantTFDB (http://planttfdb.cbi.pku.edu.cn/index.php;Jin *et al.*, 2014). Due to the large number of sequences that could bind to the ABREs found in the *DcPSY2* promoter, in this work we selected only sequences with homology to bZIP transcription factors of group A, specifically to the subfamily of Arabidopsis transcription factors named AREB/ABF, because the members of this subfamily have been shown to bind to ABREs and activate the expression of genes in response to ABA in other species (Fujita *et al.*, 2005; Furihata *et al.*, 2006; Kim, 2006). After the alignment, the three sequences that showed the highest identity to the AREB/ABF subfamily of transcription factors were selected, and were named *DcAREB1*, *DcAREB3*, and *DcAREB4*. To identify conserved domains present in the sequences, we used the NCBI CD-Search tool; all three sequences possess all the domains of the AREB/ABF subfamily (see Supplementary Fig. S2). The amino acid identity between the selected sequences and the Arabidopsis AREB/ABF transcription factors suggests that *DcAREB1* and *DcAREB4* could encode ABRE binding proteins involved in the signal transduction of ABA-regulated genes during maturation and seed development (Bensmihen *et al.*, 2002, 2005; Kim *et al.*, 2002; Kim, 2006; Lopez-Molina and Chua, 2000), while *DcAREB3* could encode an ABRE binding protein involved in the ABA regulation of genes in vegetative tissues in response to abiotic stress (Choi *et al.*, 2000; Uno *et al.*, 2000; Kang *et al.*, 2002; Kim *et al.*, 2002; Fujita *et al.*, 2005). The subcellular localization of these three *D. carota* AREB/ABF transcription factors was determined by means of a double transient transformation of tobacco leaves with vectors carrying the sequences of *DcAREB1*, *DcAREB3*, and *DcAREB4* fused to *RFP* and the pCAMBIA1302 vector, which possesses the *GFP* gene directed by the constitutive promoter 35SCaMV, with nuclear GFP localization (Marker Gene Technologies, Inc.). Figure 5 shows that the three *D. carota* AREB/ABF transcription factors are localized in the nucleus. The subcellular localization of DcAREB1, DcAREB3, and DcAREB4 was also confirmed through carrot protoplast transfection (Supplementary Fig. S3).

**Fig. 6.**
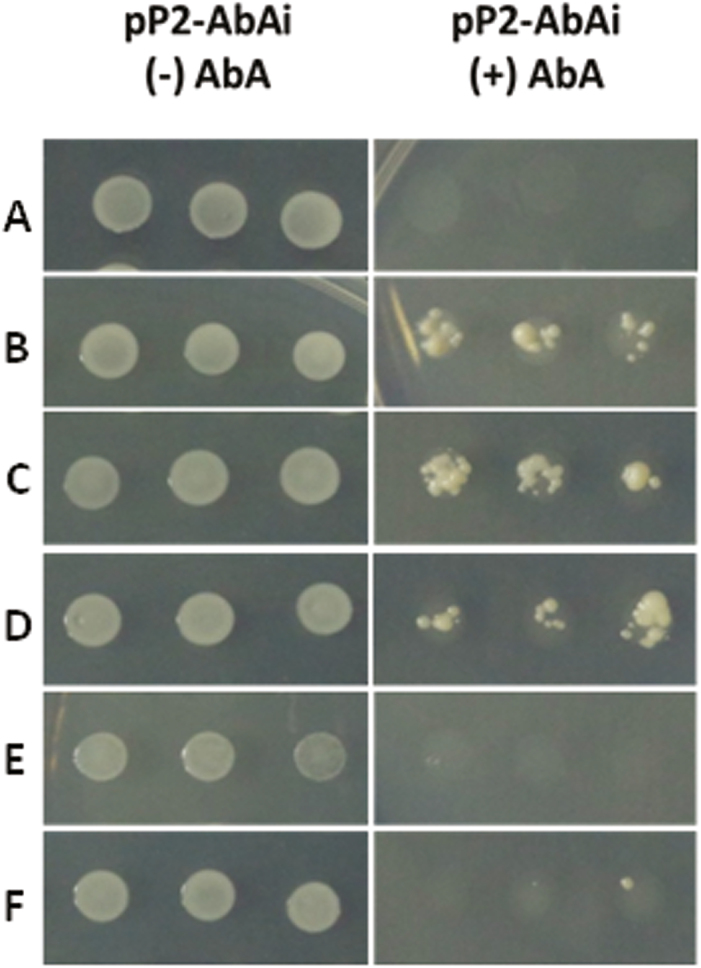
Binding of DcAREB transcription factors to the *DcPSY2* promoter. A monohybrid assay was performed with the Y1HGold-pP2-AbA1 (pP2-AbAi) yeast strain transformed with the pDEST22-FT vectors that express *DcAREB1*, *DcAREB3*, or *DcAREB4*. Transformed yeast strains were grown on SD/−Ura−Trp medium supplemented with or without Aurobasidin A (AbAi). (A) pDEST22 empty vector; (B) pDEST22 DcABRE1; (C) pDEST22 DcABRE3l (D) pDEST22 DcABRE4; (E) pDEST22 CAREB1; (F) pDEST22 CAREB2.

In order to determine the ability of the three *D. carota* AREB/ABF transcription factors to bind to the *DcPSY2* promoter, we performed a yeast one-hybrid (Y1H) assay. The *Saccharomyces cerevisiae* Y1HGol-pP2-AbAi reporter strain was generated using the vector pAbAi, in which the Aureobasidin A antibiotic resistance gene, *AUR1-C*, is directed by the *D. carota DcPSY2* P2 promoter. These reporter strains were transformed with the pDEST22 vectors carrying the sequences of the three *D. carota* AREB/ABF transcription factors fused to the activation domain (AD) of the GAL4 protein. Figure 6 shows that the reporter strain transformed with the empty vector pDEST22 is not able to grow in a medium supplemented with the antibiotic Aureobasidin A. However, the reporter strain transformed with the pDEST22 vectors that carry the sequences *DcAREB1*, *DcAREB3*, or *DcAREB4* fused to GAL4 AD grow in the presence of Aureobasidin A (Fig. 6). As a negative control, the same yeast strain was transformed with a vector carrying the sequences of *CAREB1* or *CAREB2* fused to GAL4 AD, which encode bZIP transcription factors of *D. carota* that are induced by ABA, but recognize another ABRE sequence, different from that found in the promoter of *DcPSY2* (Guan *et al.*, 2009). As expected, the transformed reporter strain was unable to grow in the presence of Aureobasidin A (Fig. 6). These results suggest that, at least in yeast, DcAREB1, DcAREB3, and DcAREB4 bind specifically to the *DcPSY2* promoter.

**Fig. 7.**
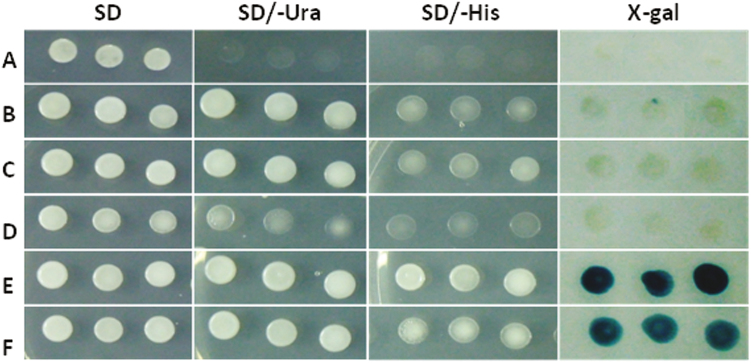
Transactivation assay of DcAREB transcription factors. Expression of the reporter genes *URA3*, *HIS3*, and *lacZ* in the yeast strain MaV203 transformed with the pDEST32-FT constructs and grown on SD (SD/−Leu medium, SD baseline without leucine), SD/−Ura (auxotrophy for uracil), SD/−His + 25 mM 3-amino-1,2,4-triazole (auxotrophy for histidine). The X-gal column corresponds to the assay performed to examine the induction of lacZ. (A) pDEST22 empty vector; (B) pDEST22 DcABRE1; (C) pDEST22 DcABRE3; (D) pDEST22 DcABRE4; (E) pDEST22 CAREB1; (F) pDEST22 CAREB2.

Similarly, to determine if the three *D. carota* transcription factors could activate transcription by themselves, we performed an Y1H assay using the *S. cerevisiae* strain MaV203 (Vidal *et al.*, 1996). The MaV203 strain contains three reporter genes (*URA3*, *HIS3*, and *lacZ*) directed by promoters that include binding sites for the DNA-binding domain (DBD) of the GAL4 protein. This reporter strain was transformed with the pDEST32 vectors carrying the sequences of the three *D. carota* AREB/ABF transcription factors fused to the GAL4 DBD. The reporter strain transformed with the empty vector pDEST32 was unable to grow in synthetic defined medium supplemented with all amino acids except uracil or histidine. However, the reporter strain transformed with the vectors that carry the *D. carota* transcription factors fused to the GAL4 DBD grew in a medium without uracil or histidine (SD/−Leu−Ura and SD/−Leu−His supplemented with 25 mM 3-amino-1,2,4-triazole, respectively) (Fig. 7), indicating that these transcription factors induce the expression of the reporter genes *URA3* and *HIS3.* Additionally, an X-gal assay for these strains showed a faint blue color. As positive control, we included the CAREB1 and CAREB2 sequences that were proven previously to be functional transcription factors (Guan *et al.*, 2009). These results confirm the function of DcAREB1, DcAREB3, and DcAREB4 as transcription factors.

**Fig. 8.**
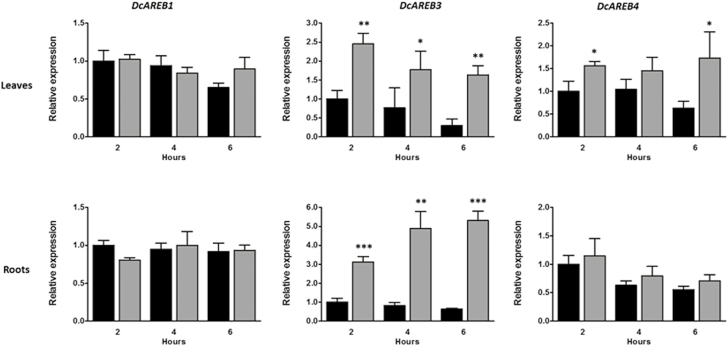
Relative expression of *DcAREB* transcription factors under ABA treatment. (A) Normalized transcript levels of *DcAREB1*, *DcAREB3*, and *DcAREB4* in leaves or roots of 4-week-old carrot plants after 2, 4, and 6 h of water (black bars) or 100 µM ABA treatment (gray bars). Transcript abundance was normalized to *ubiquitin* gene expression level and the control condition was taken as calibrator. All values represent the means of three independent values (±SD). Asterisks indicate statistically significant differences using an unpaired two-tailed *t*-test. *P < 0.05, **P < 0.01, ***P < 0.001.

To complement these results, we performed an expression analysis of *DcAREB1*, *DcAREB3*, and *DcAREB4* in 4-week-old carrot seedlings under ABA treatment. Figure 8 shows that after treatment with ABA for 2, 4, and 6 h, *DcAREB1* is not induced, either in leaves or in roots; *DcAREB4* is induced in leaves but not in roots; and *DcAREB3* is highly induced in both organs. These results suggest that *DcAREB3* encodes an ABRE binding protein capable of regulating genes in response to ABA in vegetative tissues.

## Discussion

### *DcPSY1* and *DcPSY2* respond differently to salt stress and ABA in carrot

Two carrot paralog *PSY* genes are expressed differentially in leaves and roots during plant development (Fuentes *et al.*, 2012; Simpson *et al.*, 2016), with *DcPSY1* being mostly expressed in leaves and *DcPSY2* in the storage root. Interestingly, although they share 68.34% identity at the amino acidic level, DcPSY1 is more closely related to PSYs of monocot species whereas DcPSY2 is most closely related to those of dicots (Qin *et al.*, 2011), providing evolutionary support for the putative differential role of these genes in carotenoid synthesis in carrot. NaCl treatment of carrot seedlings induces the expression of both *PSY1* and *PSY2* in leaves and roots (Fig. 2A), and produces an increase in ABA (Fig. 2D, F), suggesting that salt stress increases ABA levels in the whole plant through the expression of *PSY* genes. However, the fact that acute salt stress results in a much higher induction of *DcPSY2* expression compared with *DcPSY1* (Fig. 2A), and that only *DcPSY2* transcript abundance rises after ABA treatment, preferably in roots (Fig. 2F), suggests a differential expression mechanism and *in vivo* function of both genes. It is possible that *DcPSY1* may respond to salt stress by an ABA-independent transduction mechanism that is sustained by the absence of enough ABRE elements in the *DcPSY1* promoter (Fig. 3). Different expression patterns of *PSY* paralog genes have been demonstrated in other plants such as maize, rice, and tomato. In tomato, *SlPSY1* is expressed mainly in fruits, *SlPSY2* is more prevalent in leaves and flowers, and *SlPSY3* is predicted to respond to abiotic stress (Bartley and Scolnik, 1993; Fraser *et al.*, 1999; Giorio *et al.*, 2008). In maize and rice, the *PSY3* paralog responds to ABA treatments while *PSY1* and *PSY2* are induced by light (Li *et al.*, 2008; Welsch *et al.*, 2008). This supports the fact that PSY paralogs are able to display functional specificity and diversity in plants.

### Identification and characterization of the *DcPSY2* promoter of *Daucus carota*

Considering that ABA activates the expression of *DcPSY2* in carrot, we isolated the promoter of *DcPSY2* to identify possible ABREs, also taking into account that the regulation of the carotenoid biosynthetic pathway occurs mainly through the control of *PSY* expression. It is important to note that the sequence of the *DcPSY2* promoter obtained by genome walking is 98% identical to the one annotated in the recently sequenced carrot genome (Iorizzo *et al.*, 2016). The promoter presents several *cis*-responsive elements and most of them are related to light (light responsive elements), which can be explained by the absolute requirement of carotenoids during photosynthesis and for the dissipation of excess light energy (Demmig-Adams *et al.*, 1996; Niyogi, 1999; Pogson *et al*., 2005; Demmig-Adams and Adams, 2006; Dall’Osto *et al.*, 2010; Stange and Flores, 2012). Carotenoid biosynthetic genes such as isopentenyl isomerase (*IPI*) in maize and *PSY* in Arabidopsis are induced during de-etiolation (Albrecht and Sandmann, 1994; von Lintig *et al.*, 1997; Welsch *et al.*, 2000; Pizarro and Stange, 2009; Rodríguez-Villalón *et al.*, 2009*a*,*b*; Toledo-Ortiz *et al.*, 2010). Similarly, we observed that *DcPSY1* and *DcPSY2* are also induced by light in leaves during development of carrots (Fuentes *et al.*, 2012).

The promoter also possesses *cis*-responsive elements for hormones such as auxin, gibberellin, and ABA, suggesting a role of *DcPSY2* during plant growth. The relationship of hormones with carotenoid biosynthesis has been reported previously. Kraft *et al.* (2007) showed that auxin treatments induce the expression of *NCED*. Additionally, gibberellin synthesis shares the isoprenoid precursor geranyl geranyl pyrophosphate (GGPP) with the carotenoid pathway, and the overexpression of carrot *LCYB1* in tobacco produces an increase in carotenoids, gibberellins, and chlorophylls, as well as the expression of key genes of each biosynthetic pathway (Moreno *et al.*, 2016). With respect to ABA responses, three ABREs were found in the isolated promoter, two of them in sufficient proximity (27 bp apart) to form an ABRC (Shen *et al.*, 1996). The ABRE motives are consistent with those found in the complete *DcPSY2* promoter in the recently sequenced carrot genome (Iorizzo *et al.*, 2016). The ABREs are recognized by transcription factors of the bZIP family (Guiltinan *et al.*, 1990; Hobo *et al.*, 1999; Choi *et al.*, 2000; Uno *et al.*, 2000), such as ABI5 and ABI3, which are induced by ABA in the root of Arabidopsis (Brocard *et al.*, 2002; Brocard-Gifford *et al.*, 2003). The overexpression of the bZIP transcription factors ABF3 or ABF4 in Arabidopsis alters the expression pattern of genes regulated by ABA and confers tolerance to abiotic stress (Kang *et al.*, 2002; Kim *et al.*, 2004). In carrot, the transcription factors C-ABI3, CAREB1, and CAREB2 have been described as being involved in the response to ABA during carrot somatic embryogenesis (Shiota *et al.*, 1998; Guan *et al.*, 2009), but no AREB/ABF transcription factors have been studied in abiotic stress response and carotenoid synthesis until now.

### Identification and functional characterization of AREB/ABF transcription factors in *Daucus carota*

In plants, most of the physiological responses to ABA occur through transcriptional regulation (Busk and Pagès, 1998) mediated by transcription factors of the bZIP family (Hobo *et al.*, 1999; Choi *et al.*, 2000; Uno *et al.*, 2000). Specifically, those of group A represented by the AREB/ABF transcription factors (Choi *et al.*, 2000; Uno *et al.*, 2000) bind to ABREs *in vitro* and induce the expression of ABA responsive genes, like *osmotin* (Fujita *et al.*, 2005; Furihata *et al.*, 2006; Kim, 2006). ABF3 and AREB1/ABF2 bind to ABREs in Arabidopsis and are activated by drought, salinity, and ABA treatments (Fujita *et al.*, 2005), and their overexpression results in increased drought tolerance (Kang *et al.*, 2002; Kim *et al.*, 2004; Fujita *et al.*, 2005). In this work, three AREB transcription factors (DcAREB1, DcAREB3, and DcAREB4) were functionally characterized in carrot. All of them showed nuclear localization and direct binding to the *DcPSY2* promoter and transactivate reporter genes in yeast monohybrid systems. As expected, CAREB1 and CAREB2 were not able to bind to the *DcPSY2* promoter, allowing us also to conclude that the predicted *DcPSY2* promoter ABRE elements (TATGTG, CACTTG, CGTGG) are not recognized by CAREB1 or CAREB2. The lower β-galactosidase activity observed in the transactivation assay for DcAREB1 and DcAREB3 (Fig. 7) was probably due to the fact that these transcription factors usually form heterodimers *in vivo*. The bZIP domain is composed of a bipartite α-helix at the N-terminal region formed by basic amino acids capable of interacting with the major groove of DNA in a sequence-specific manner and an amphipathic α-helix at the C-terminus that is required for the dimerization with the bZIP domain from another transcription factor, termed a leucine zipper (Vinson *et al.*, 2006; Llorca *et al.*, 2014). Transcription factors that belong to the G class of bZIPs, such as CAREB1 and CAREB2, possess a proline domain that directs transactivation in a monomeric state (Miotto and Struhl, 2006; Shen *et al.*, 2008), while others, such as those of group A (including the three DcAREBs analysed here), require additional elements as co-activators (Rochon *et al.*, 2006) or modifications (Kuo *et al.*, 2000). Such information is relevant in helping to understand the differences in transactivation of DcAREB1, DcABRE3, and DcABRE4 with respect to that of CAREB1 and CAREB2. In addition, the transactivation capacity of bZIP transcription factors can be modified through the interaction with other proteins (Andronis *et al.*, 2008; Gangappa *et al.*, 2013). In the case of DcAREB4, the transactivation activity appeared to be even less than for DcAREB1 and DcAREB3 (Fig. 7). Interestingly, DcAREB4 does not possess the dimerization domain at the C-terminus that is present in the other transcription factors (see Supplementary Fig. S2). Thus, the absence of the dimerization domain in DcABRE4 could impair the formation of stable dimers required for the proper activation of the reporter genes. Therefore, during an *in vivo* response, the composition of heterodimers determines the expression of the target gene (Llorca *et al.*, 2014).

The expression patterns of *DcAREB1*, *DcAREB3*, and *DcAREB4* in response to ABA treatment in carrot suggest that DcAREB3 may be responsible *in vivo* for ABA-mediated abiotic stress tolerance in carrot roots (Fig. 8), correlating with the function described for *AREB3* in Arabidopsis and the expression of *DcPSY2* in carrot after ABA and salt treatment (Fig. 2F).

**Fig. 9.**
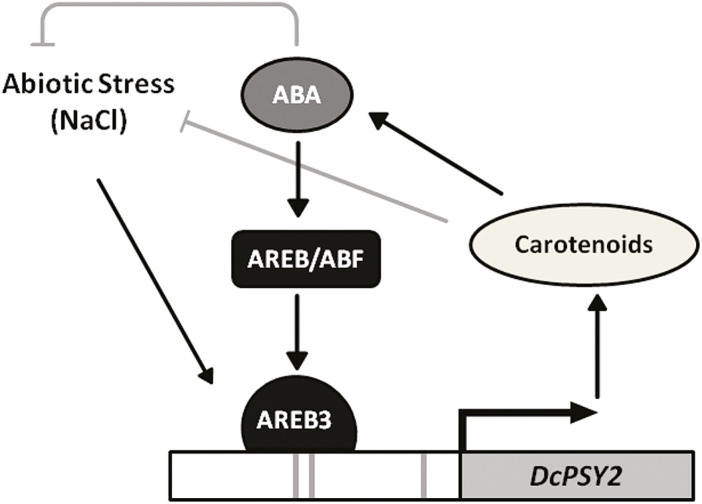
Proposed model that summarizes the induction of *DcPSY2* expression by ABA during salt stress treatment. Salt stress increase *DcPSY2* expression and carotenoid and ABA levels in carrot roots during abiotic stress tolerance. In addition, ABA induce the expression of *DcPSY2* as a positive feedback mechanism accomplished by the direct binding of DcAREB transcription factors, such as DcAREB3, to the *DcPSY2* promoter.

The discovery of DcAREB3 and its ability to bind to the *DcPSY2* promoter allows for a better understanding of how abiotic stress increases ABA levels in plants, and how ABA and increased carotenoid levels are linked to respond to this kind of stress in roots. Taken together, we propose a model (Fig. 9) that may reflect the *in vivo* response to salt stress in carrot seedlings and an explanation of how ABA is able to regulate the synthesis of their metabolic precursors through the increase in the expression of a key carotenogenic gene, *PSY2*. In this model, salt stress and ABA induce the expression of *DcPSY2* (preferably in roots) through the binding of AREB transcription factors (probably DcAREB3) to the ABREs found in the promoter of *DcPSY2*. The rise in the expression of *DcPSY2* in carrot roots increases the production of carotenoids and consequently ABA levels increased, protecting the plant against abiotic stress. It is important to highlight that carotenoids themselves can also provide aid under stress conditions due to their antioxidant properties. Therefore, carotenoids are required in plant roots to respond to osmotic stress. In leaves, the production of ABA mostly relies on the cleavage of available xanthophylls correlating with the induction of *NCED* genes (Ruiz-Sola *et al.*, 2014). Therefore, salt stress increased ABA levels in carrot leaves without an increase in carotenoids, although *DcPSY2* and *DcAREB3* were induced (Fig. 2B, D), possibly due to the high activity of β,β branch enzymes. Thus, an enhanced expression of β,β branch genes might contribute to convert efficiently β-carotene into xanthophylls that NCED3 cleaves to produce ABA (Ruiz-Sola *et al.*, 2014).

Carotenoids are usually scarce in roots (including the roots of carrot seedlings), and therefore it is not surprising that in diverse models, such as Arabidopsis, maize, and rice, the *de novo* biosynthesis of ABA in roots involves the tissue-specific induction of key carotenogenic genes, particularly *PSY* (Li *et al.*, 2008; Welsch *et al.*, 2008; Li *et al.*, 2009; Ruiz-Sola *et al.*, 2014). Similar to these previously reported findings, the induction of *DcPSY2* in carrot roots after salt stress is accompanied by an increase in total carotenoids in this tissue, indicating that the decrease in ABA precursors is compensated by the induction of *DcPSY2* expression. Although the accumulation of ABA in dehydrated citrus roots depends mainly on the transport from aerial organs (Manzi *et al.*, 2015, 2016), our results support the idea that ABA is able to induce the synthesis of its own metabolic precursors through the up-regulation of carotenogenic genes in roots of carrot seedlings. On the other hand, different studies demonstrate that AREB/ABFs are master transcription factors that regulate ABRE-dependent ABA signaling during osmotic stress, while their overexpression enhances drought tolerance (Uno *et al.*, 2000; Kang *et al.*, 2002; Kim *et al.*, 2004; Fujita *et al.*, 2005, 2011; Yoshida *et al.*, 2010; Li *et al.*, 2013). To date and to our understanding, this is the first report that links the AREB/ABF transcription factor family to the induction of carotenoid production in response to ABA in plants.

## Supplementary data

Supplementary data are available at *JXB* online.

Fig. S1. DcPSY2 promoter sequence.

Fig. S2. Schematic representation of DcAREB transcription factors.

Fig. S3. Subcellular localization of DcAREB transcription factors in carrot protoplasts.

Table S1. Primers used in this work.

Table S2. Predicted regulatory motifs in the *DcPSY2* promoter.

Supplementary Figures and Tables
