# Supplementary material for: Unraveling the induction of phytoene synthase 2 expression by salt stress and abscisic acid in Daucus carota
Source: J Exp Bot. 2018 May 30;69(16):4113–26. doi: 10.1093/jxb/ery207 (PMC6054239; doi:10.1093/jxb/ery207)
Supplement: Supplementary Figures and Tables [file ery207_suppl_supplementary_figures_tables.pdf]

Unraveling the induction of phytoene synthase 2 (DcPSY2) expression by salt stress and abscisic acid in Daucus carota.

Simpson K[1], Fuentes P[1,2], Quiroz-Iturra L.F[1], Flores-Ortiz C[1], Contreras R[3], Handford M[1] and Stange C[1].

1:Laboratorio de Biología Molecular Vegetal, Departamento de Biología, Facultad de Ciencias, Universidad de Chile, Las Palmeras 3425, Casilla 653, Ñuñoa, Santiago, Chile.

2: Current address: Max Planck Institut für Molekulare Pflanzenphysiologie, Potsdam-Golm, Germany

3:Laboratorio de Fisiología y Biotecnología Vegetal, Facultad de Química y Biología, Universidad de Santiago de Chile, Santiago, Chile.

E-mail: cstange@uchile.cl

5'-
AAACCCCAAAAATACTTGCCTGTATATTCTTCATACAGTGGTATAAAAACTGGATCAATATCCCCAGACCAGAAAAAACCCAAAAAGAAATGTGTATATATATGCAAGA
GAAGTTGTAAAATGAAAGAGTAAAACAAGAGAAGCCGGAAGGGGAATCTTGAAGAGCTAAGATTTGTGGGTCACAATCTTAGCCAATTCTTCTGTTGTTTATTTTTATA
AATAAATAATATTAATTAATTTATATGATGAGATAGTGATAATGATGATGATATTGTGGAGCTTGTTGGTGTGAATGGTTGAGGCCTCTCCACTGAGGTTAGTTTTTTTCT
CAGCTCTTCTTTTCCTTGTCTTCTTTCAATTCATTGATTATGTTTCTCACACTATATACTGTTACACTCATCTATGTGTATATATAAT**AGCGT**TGGTTGTTTGGATTTTG**A**AT
TTCACTTGTGGCCACTATGCAAAGTTGAGCTTAATAGTAGCaaacacccattgaatcccaattcccaaataagctttttgttacatgctcttctttgtttcttcatagttgatacatatatcatttgtattcttcagttgttttt
gatattccttttatatattttctacagaatttcttaggaaagaaagaagctttactgccacttaattattgactttgctttgacttggtttcattttagttatatatcttcttttattttgaattaggatttgttcttagctaataaact**tccgtg**ggtgttctattttt
ctcataattgaagctcgccggaaaaagacaaaccaaaggaggttattgaattgaagtttattgggtagcactaactccagc-3'

Fig S1. DcPSY2 promoter sequence. The 769 bp fragment obtained by genome walking is shown. In lower case: 5'UTR, bold A: TSS (transcription start site), underlined: putative TATA box.

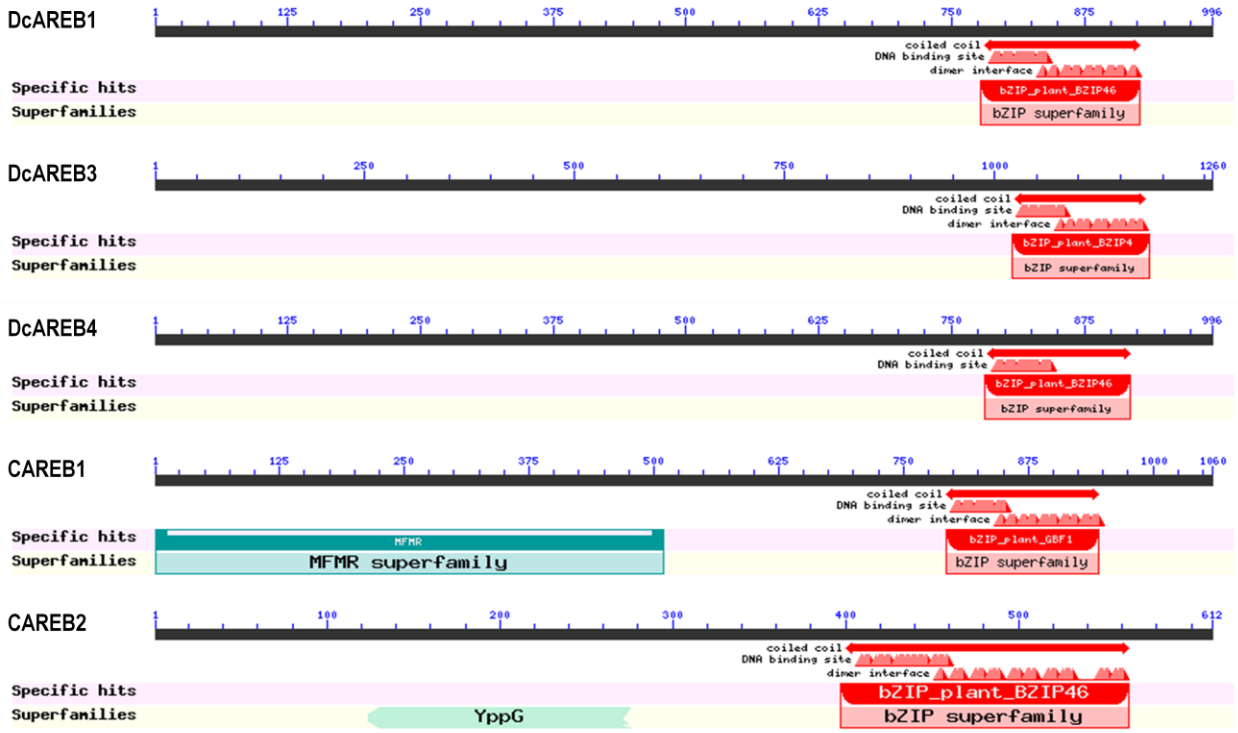

Fig S2. Schematic representation of DcAREB transcription factors. b-ZIP conserved domains in the DcAREB transcription factor candidates were identified using the CD-Search platform. The double-headed red arrow shows the presence of a coiled coil structural motif. A possible DNA binding site and a dimerization domain are also depicted. A) DcABRE1, B) DcABRE3, C) DcABRE4, D) CAREB1, E) CAREB2.

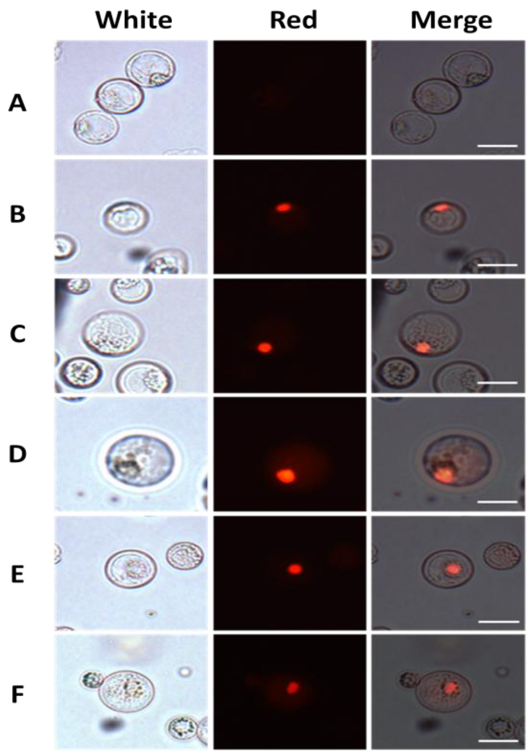

Fig S3. Subcellular localization of DcAREB transcription factors in carrot protoplasts. Carrot protoplasts were transfected with pK7RWG2 empty vector carrying the 35S:RFP construct and DcAREB:RFP vectors. Images were taken 24 h after transfection. A) pK7RWG2 empty vector, B) DcABRE1:RFP, C) DcABRE3:RFP, D) DcABRE4:RFP, E) CAREB1A:RFP, F) CAREB2:RFP. Red channel: Images taken under the Cy3 filter. Bar: 10 µm.

Table S1: Primers used in this work. Primer sequences and uses.

Name	Sequence 5´- 3´	Tm	Description
P1	GCGTTGGTTGTTTGGATTTTGAA	55,2	To amplify the P1 (421 bp) fragment of *DcPSY2* promoter together with PSY2R
P2	CAGAAAAAACCCAAAAAGAAATGTG	52,2	To amplify the P2 (769 bp) fragment of *DcPSY2* promoter together with PSY2R
PSY2R	GCTGGAGTTAGTGCTACCCAATAAAC	57,5	Used for the amplification of *DcPSY2* promoter fragments
AREB1 F	GGGCTAGTAATAGTTTGGTGTGGT	56.6	To amplify the complete cds of *DcAREB 1*
AREB 1 R	AAAAGATGCTGAGCTGGTCCTGC	59.9	
AREB 3 F	GGCTTGTCTGGATCAGGATCAGC	59.7	To amplify the complete cds of *DcAREB 3*
AREB 3 R	CCAAGGTCCGGTCAGGGTCC	62.5	
AREB 4 F	GGGTTCTGGGTTTGTGTGATAGG	57.7	To amplify the complete cds of *DcAREB 4*
AREB 4 R	GAAATCTGCTGAACTTGTCCGGCG	60.5	
qAREB1F	TCTTGGTTAAGGCGGGTGTGATTAG	58.7	To amplify a 149 bp fragment of *DcAREB1* for qRT.
qAREB1R	CTGAGTTGGCTGATGAGTCGGG	59.7	
qAREB3F	GCCAGTTTAGCACCTCGTGTTGC	60.9	To amplify a 179 bp fragment of *DcAREB3* for qRT.
qAREB3R	CGCCTTCTCTCCACCACTTTCTC	59.5	
qAREB4F	GGCAATCAAGTGTAACCTTACCTCGG	59.4	To amplify a 185 bp fragment of *DcAREB4* for qRT.
qAREB4R	TCCTCCTTCAGTAACTACCCCTGC	59.8	
q*psy2* F*	GCTAATAAACTTCCGTGGGTGTTC	53	To amplify a 112 bp fragment of *DcPSY2* for qRT.
q*psy2* R*	GCTGGAGTTAGTGCTACCC	57	
q*psy1* F	AGTCGATGGAGCATTACCATAATTC	53	To amplify a 95 bp fragment of *DcPSY2* for qRT.
q*psy1* R	CTAATGGGTTACAGAGGGTTGTGTTA	57	
qNt *osmotin* F	CTTCCTCCTTGCCTTGGTGACTTATACT	57	To amplify a 164bp of *osmotin* gene of *N. tabacum*.
qNt *osmotin* R	CGTGCCATTTTAGTACCTCGTGGTGCAT	60	
HptII F	TTTGTGTACGCCCGACAGT	52	Hygromicin resistance gene. To check *N. tabacum* DcPSY2 P2:GFP and DcPSY2P1:GFP transgenic plants
HptII R	AAGACCTGCCTGAAACCGA	58	
18S F	TTGATTACGTCCCTGCCCTTT	52	Used in qRT as *N. tabacum* housekeeping gene.
18S R	ACAATGATCCTTCCGCAGGT	60	
ubiq F	GCTCGAGGACGGCAGAAC	53	Used in qRT as carrot housekeeping gene.
ubiq R	CTTGGGCTTGGTGTAGGTCTTC	60	
GW1*PSY2*	TAACCTCCTTTGGTTTGTCTTTTTCCGGCG	63,2	*DcPSY2* antisense specific primer for first PCR in GenomeWalking
GW2*PSY2*	GCTTATTTGGGAATTGGGATTCAATGGGT G	60,6	*DcPSY2* antisense specific primer for nested PCR in GenomeWalking
AP1	GTAATACGACTCACTATAGGGC	51,9	AP-specific binding linker for first PCR in GenomeWalking
AP2	ACTATAGGGCACGCGTGGT	57,8	AP-specific binding linker for nested PCR in GenomeWalking

Table S2: Predicted regulatory motifs in the *DcPSY2* promoter. Regulatory elements that respond to different stimuli and hormones are included for P2 and P1 *DcPSY2* promoter fragments.

Response to:	Cis element name	N° of cis elements in P2	N° of cis elements in P1	Description
**Light**	*G-Box*	4	3	Light Responsive element (LRE)
	Others	51	23	Light Responsive element (LRE)
**TATA/**	*TATA*	9	1	TATA
**Enhancer**	*CAAT* box	4	2	Enhancer
	5UTR_Py-rich_stre	1	0	Confers high transcription
**Repressors**	*ACI*	1	1	Negative regulator in phloem
	ACII	1	0	Negative regulator in phloem
	Motivo AACA	0	0	Repressor in endosperm
**Biotic Stress**	*JERE*	1	1	Response to elicitors, wounding and pathogens
	Box W	2	2	Response to elicitors, wounding and pathogens
	Box S	0	0	Response to elicitors, wounding and pathogen
	ELI-box3	2	1	Response to elicitors
	Motivo WUN	0	0	Response to wounding
	EIRE	0	0	Response to elicitors
	Box W1	2	2	Response to fungal elicitors
	Box E	1	0	Response to fungal elicitors
**Abiotic stress**	*DRE*	0	0	Drought, low T°, salinity
	MBS	0	0	Drought
	HSE	2	2	Heat-shock
	LTR	1	0	Low T°
	ARE	1	0	Anaerobic induction
	GC motif	0	0	Anoxic induction
**Abscisic acid (ABA)**	*ABRE*	3	2	ABA responsive element
	CE1	0	0	Coupling element for ABA response
	CE3	0	0	Coupling element for ABA response
	IIb motif	0	0	ABA responsive element
	I & IIa motif	0	0	ABA responsive element
**Auxin**	*TGA*	2	2	Auxin responsive element
	GATA Box	1	1	Absence of Auxin responsive element
	AuxRE	1	1	Auxin responsive element
	AuxRR-core	0	0	Auxin responsive element induced by stress
	as-1 box	0	0	Auxin, salicylic acid and methyl

				jasmonate responsive element
**Gibberellin**	*GARE motif*	2	1	Gibberellin responsive element
	Box P	1	1	Gibberellin responsive element
	Box TATC	1	1	Gibberellin responsive element
**Salicylic acid (SA)**	*TCA element*	1	0	SA responsive element
**Methyl Jasmonate**	*CGTCA motif*	0	0	Methyl jasmonate responsive element
	TGACG motif	2	1	Methyl jasmonate responsive element
**Ethylene**	*ERE*	0	0	Ethylene responsive element
